# Designing online health services for patients

**DOI:** 10.1186/s13584-016-0082-7

**Published:** 2016-06-15

**Authors:** Bradley H. Crotty, Warner V. Slack

**Affiliations:** Division of Clinical Informatics, Beth Israel Deaconess Medical Center and Harvard Medical School, 330 Brookline Avenue, Boston, MA 02115 USA

**Keywords:** Patient portal, Digital health, Telehealth, Personal health record, Consumer informatics

## Abstract

Patients are increasingly interacting with their healthcare system through online health services, such as patient portals and telehealth programs. Recently, Shabrabani and Mizrachi provided data outlining factors that are most important for users or potential users of these online services. The authors conclude convincingly that while online health services have great potential to be helpful to their users, they could be better designed. As patients and their families play an increasingly active role in their health care, online health services should be made easier for them to use and better suited to their health-related needs. Further, the online services should be more welcoming to people of all literacy levels and from all socioeconomic backgrounds.

## Background

Increasingly, people are going online for help with their own health or help with the health of a family member; and increasingly, health care providers in turn are offering patients and prospective patients online services by means of portals that enable them to schedule their appointments, view their health records and interact with their clinicians [1]. Although still nascent, telehealth solutions that deliver care through video conversations are promising to help connect patients with healthcare on their own terms [2]. And companies are creating and offering online approaches to health promotion directly to patients, such as health and fitness tracking, laboratory procedures and medical record keeping [3]. In Israel, the health plans (also known as health maintenance organizations or HMOs), have been an important driver in promoting technology to help to engage patients [4].

## Decision to use online health services

Knowing why people use or do not use online health services is important information for health care facilities as they plan for the future. In a recently published IJHPR article, Shahrabani and Mizrachi share data they collected from over 700 randomly sampled Israelis by a telephone survey [5]. Their focus was on individuals aged 45 years or older. Their survey asked about the use of, and attitudes towards, online health services (OHS), with an emphasis on services offered through Israeli health maintenance organizations.

Of the 59 % of respondents who were Internet users, more than three quarters had used at least one OHS. The data showed differences in use by culture, religion and education. Of the Internet users who were not using an OHS, the most common reasons given were that the online services were not easy to use or did not meet a particular need. As one might expect, older participants more commonly noted that the sites were difficult to use. The next most common reason was that the survey respondent either did not know about the existence of such services or had not been encouraged to use them. Further, the results of the survey provide an important snapshot of approaches to portals that are similar to those from other developed countries, where higher education and literacy levels are associated with increased adoption [6].

## Increasing adoption and usage through thoughtful design

On the basis of their results, the authors recommend that health maintenance organizations do more to advertise and otherwise encourage the use of their online health services to help increase adoption. Our experience in Boston suggests that system-wide encouragement of use of portals will lead to increased adoption. In 2012, when we made concerted efforts to advertise our portal services to patients and to more actively encourage adoption, our rate of growth of online patients tripled.

We believe, however, that the data from Shahrabani and Mizrachi have an even more important lesson. Online health services need to be designed better for their use by patients. Although the goals of healthcare organizations are for the most part aligned with the needs of their patients, such as providing up-to-date information together with tools to help in healthcare management, the human factors of the computer displays are often confusing and the requirements for interacting with the computer are often difficult for the patient to master.

Online health services must be intuitive to use. Developers of online systems should adopt user-centered design, when possible drawing upon experience among people with different ages, different abilities and different levels of education and literacy [7]; studies in Israel and the United States show that literacy and “eliteracy” are important for deriving benefits from contemporary portals [8]. Medical terminology can be confusing and intimidating, and online health services have an amazing opportunity to assist people with their health literacy by several means, such as by educational links. Online services can also be translatable into languages more suitable for minority groups among the users. Further, online services can be helpful in enhancing communication among family members, with shared access for health-care proxies—whether they be family members or non-family members—to assist with the management of health needs and communication with clinicians [9].

The needs of patients are evolving. While the users in this survey turned to their online health services most often for help with administrative tasks – and this is an important first start – there are many opportunities to engage patients further with their healthcare by opening up new avenues of communication [10]. The OpenNotes movement at Beth Israel Deaconess Medical Center and collaborating institutions, which encourages clinicians to share their medical record notes openly with patients, has resulted in patients feeling they have more control over their healthcare and are better equipped to follow-through with their medications [11, 12]. Portals can also help patients prepare for visits by reviewing their records and learning about and addressing gaps in their care when they occur. Modules available through portals, such as one we recently created for educating patients about advance directives, can serve the dual purpose of providing information to patients and helping organizations in turn to collect important clinical information for their records [13].

Mobile platforms, such as smart phones and tablets, are rapidly replacing traditional computers as the primary means for people to access the Internet, particularly those from lower socioeconomic status. Additionally, older people may find interacting with a tablet an easier experience than navigating with a mouse and computer. This trend has important implications for system design. Online health services that don’t accommodate smart phone and tablet use will fail to meet the needs of a sizeable number of patients.

## Conclusions

Shahrabani and Mizrachi’s survey has provided an important service. Their results remind us convincingly that ease of use, especially for older patients, is a major driver of new technology [14]. When designing online health services, the needs of the patient should come before the needs of the organization, and usability should be targeted for a large, diverse range of patients and not limited to those who are already comfortable with new technology.

We are at the beginning of learning how best to engage patients with online services. Our tools are still rather primitive, though we hope they will improve over time. Ongoing research is needed to learn how clinicians can best encourage patients to sustain their engagement in their health and healthcare, with support from online technology. Further, rather than treating ‘electronic health records’ and ‘personal health records’ as separate entities, investigators and their clinician colleagues need to develop the means to combine them, incorporating patient generated information together with clinician generated information into a unified electronic health record, separate from financial and other administrative processes and available online for use by both patients and clinicians.

It has long been our premise that patients or prospective patients are the largest, yet least well utilized healthcare resource [15]. Online health services, when used wisely and well, can enable patients to become more integrally part of their health care system and thereby help both patients and their clinicians to improve the quality of the care provided.

## Abbreviations

HMO, Health Maintenance Organization; OHS, Online Health Services
